# How mechanics of individual muscle-tendon units define knee and ankle joint function in health and cerebral palsy—a narrative review

**DOI:** 10.3389/fbioe.2023.1287385

**Published:** 2023-12-05

**Authors:** Cemre Su Kaya Keles, Filiz Ates

**Affiliations:** Experimental Biomechanics Group, Institute of Structural Mechanics and Dynamics in Aerospace Engineering, University of Stuttgart, Stuttgart, Germany

**Keywords:** joint isometric/isokinetic torque, moment arm, muscle cross-sectional area, muscle force, muscle volume, cerebral palsy, ankle-foot deformity, knee joint pathology

## Abstract

This study reviews the relationship between muscle-tendon biomechanics and joint function, with a particular focus on how cerebral palsy (CP) affects this relationship. In healthy individuals, muscle size is a critical determinant of strength, with muscle volume, cross-sectional area, and moment arm correlating with knee and ankle joint torque for different isometric/isokinetic contractions. However, in CP, impaired muscle growth contributes to joint pathophysiology even though only a limited number of studies have investigated the impact of deficits in muscle size on pathological joint function. As muscles are the primary factors determining joint torque, in this review two main approaches used for muscle force quantification are discussed. The direct quantification of individual muscle forces from their relevant tendons through intraoperative approaches holds a high potential for characterizing healthy and diseased muscles but poses challenges due to the invasive nature of the technique. On the other hand, musculoskeletal models, using an inverse dynamic approach, can predict muscle forces, but rely on several assumptions and have inherent limitations. Neither technique has become established in routine clinical practice. Nevertheless, identifying the relative contribution of each muscle to the overall joint moment would be key for diagnosis and formulating efficient treatment strategies for patients with CP. This review emphasizes the necessity of implementing the intraoperative approach into general surgical practice, particularly for joint correction operations in diverse patient groups. Obtaining *in vivo* data directly would enhance musculoskeletal models, providing more accurate force estimations. This integrated approach can improve the clinicians’ decision-making process and advance treatment strategies by predicting changes at the muscle and joint levels before interventions, thus, holding the potential to significantly enhance clinical outcomes.

## 1 Introduction

Skeletal muscle is a specialized contractile tissue that generates force and as a fundamental mechanical consequence, it provides joint movement. The ability of a muscle to produce force and how much of its capacity is used during certain activities are central for interpreting the resulting joint function. This knowledge is essential for designing effective rehabilitation programs, tailoring exercise routines for treatment, or improving overall performance. When viewed from a different perspective, understanding 1) the extent to which neurological conditions contribute to muscle deterioration and 2) the degree to which restricted joint movement in patients with neurological, neuromuscular, or musculoskeletal diseases is attributed to muscle degradation, are also keys to make a clear diagnosis and then plan an efficient treatment.

Joint range of motion (ROM) and torque production rely on the force-generating potential of muscles that span them. The ability of a muscle to move a limb depends primarily on the length of its constituent fibers and moment arm (Delp and Maloney, 1993; Oatis, 2009). The moment arm defines the perpendicular distance between the muscle and the joint center of rotation (Rassier et al., 1999); thus, it directly depends on the location of the muscle-bone attachment side. Any alterations in muscle architecture and/or changes in the geometrical orientations between the muscles and bones (e.g., due to the shortening of muscles) affect the moment arm and subsequently alter the joint torque.

At rest, muscles provide passive resistance against movement. Upon activation, they contract, leading to either static or dynamic actions at the joint (Frontera and Ochala, 2015). Static actions, characterized by the generation of isometric forces, maintain the muscle at a constant length without resulting a joint movement; whereas dynamic actions, occurring through various types of contractions (eccentric, concentric, and isometric), lead to joint movement. Beyond the muscle moment arm, several factors (e.g., length, contraction velocity, history and level of activation (Huijing, 1998), as well as types of muscle fibers composing the muscle) affect a muscle’s tensile force. These factors ultimately impact the resultant joint torque and limb movement (Oatis, 2009).

This study reviews the relationship between muscle-tendon biomechanics and joint function by focusing on the effects of muscle size, shape, and moment arm. Furthermore, it investigates how this relationship is altered in the context of pathology, particularly in patients with cerebral palsy (CP).

## 2 The effect of the muscle-tendon unit on joint function

Muscle force both in passive and active states, is directly linked to its size. The overall muscle size describes its three-dimensional architecture (volume), primarily determined by the number and dimension of its constituent fibers. The total cross-sectional area (CSA) of a muscle, measured perpendicular to its fibers, is referred to as the physiological cross-sectional area (PCSA). The PCSA can be estimated as the ratio of a muscle’s volume to its fascicle length, corrected with the pennation angle (Lieber and Fridén, 2000).

The torque exerted at a joint is the product of the force generated by the muscles and carried by the related tendons crossing the joint and the moment arm of the muscle-tendon unit (MTU) around the joint rotation center (Baxter and Piazza, 2014). Understanding the roles of muscle size and moment arm in determining joint torque and, consequently, muscle force production requires in-depth exploration. These aspects will be thoroughly addressed in the subsequent sections.

### 2.1 Muscle size and moment arm vs. joint torque

Numerous studies have demonstrated a clear connection between muscle volume and joint torque (as summarized in Table 1), employing magnetic resonance imaging (MRI) and ultrasonography to assess muscle properties and measure associated joint torque. The relationship between muscle volume and joint torque production is well-accepted (Fukunaga et al., 2001), which applies to the ankle joint as well. For instance, the volume of the triceps surae muscle group (i.e., calf muscles) is the greatest determinant of isokinetic plantar flexion torque (Gadeberg et al., 1999) and their size (volume and CSA) is positively correlated with maximal isometric strength (Trappe et al., 2001). Similar for the anterior compartment, the volume and CSA of dorsiflexor muscles are strongly correlated with isokinetic dorsiflexion torque as well (Gadeberg et al., 1999).

**TABLE 1:** The muscle-tendon unit parameters in relation to the relevant joint function reported for healthy adults.

Reference	Study population	Muscle tested	Muscle-tendon unit parameters			Joint function		Correlations between torque and muscle-tendon unit parameters
			Volume (cm^3^)	CSA (cm^2^)	Moment arm (mm)	Activation type	MVC torque (Nm)	
Balshaw et al. (2021)	52 healthy male, age = 25 ± 2 years	Quadriceps	1838 ± 263	90 ± 12 and 167 ± 19 (PCSA)		Isometric knee extension at 65° knee position	246 ± 42 Nm	r: 0.773 (volume) *p* < 0.001
								r: 0.697 (CSA) *p* < 0.001
								r: 0.685 (PCSA) *p* < 0.001
Baxter and Piazza (2014)	20 healthy male, age = 26.0 ± 3.5 years	Plantar flexors	1348 ± 219		53.4 ± 5.6	Isometric plantar flexion at 0° ankle position	169.4 ± 52.9	r^2^: 0.322 (volume) *p* = 0.009
								r^2^: 0.315 (moment arm) *p* = 0.010
						Isokinetic plantar flexion at 30°/s	129.6 ± 47.5	r^2^: 0.226 (volume) *p* = 0.034
								r^2^: 0.465 (moment arm) *p* = 0.001
						120°/s	92.0 ± 35.4	r^2^: 0.243 (volume) *p* = 0.027
								r^2^: 0.438 (moment arm) *p* = 0.001
						210°/s	76.1 ± 28.1	r^2^: 0.222 (volume) *p* = 0.036
								r^2^: 0.484 (moment arm) *p* = 0.001
Blazevich et al. (2009)	19 healthy adults (9 male, 10 female), age = 24.2 ± 5.7 (for male), and 21.3 ± 4.3 (for female) years	Quadriceps	2347.9 ± 497.1	97.0 ± 17.8 (PCSA of VL: 110.3 ± 22.4)	40.2 ± 3.5	Isometric knee extension at 90° knee position	177.3 ± 48.4	r^2^: 0.60 (volume) *p* < 0.01
								r^2^: 0.53 (CSA) *p* < 0.01
								r^2^: 0.50 (PCSA of VL) *p* < 0.01
								r^2^: 0.25 (moment arm) *p* < 0.05
						Isokinetic knee extension at 30°/s	168.9 ± 47.2	r^2^: 0.74 (volume) *p* < 0.01
								r^2^: 0.66 (CSA) *p* < 0.01
								r^2^: 0.51 (PCSA of VL) *p* < 0.01
								r^2^: 0.18 (moment arm) *p* > 0.05
						300°/s	82.3 ± 18.9	r^2^: 0.51 (volume) *p* < 0.01
								r^2^: 0.51 (CSA) *p* < 0.01
								r^2^: 0.39 (PCSA of VL) *p* < 0.01
								r^2^: 0.19 (moment arm) *p* > 0.05
Evangelidis et al. (2016)	31 healthy male, age = 20.6 ± 2.5 years	Quadriceps	1937.3 ± 265.1	93.8 ± 10.4		Isometric knee extension at 75°, 60°, and 45° knee position	∼255 ± 50	r: 0.84 (volume) *p* < 0.001
						Isokinetic knee extension at 50°/s	∼235 ± 40	r: 0.56 (volume) *p* < 0.01
						350°/s	∼210 ± 30	r: 0.55 (volume) *p* < 0.01
		Hamstrings	794.1 ± 122.2	48.9 ± 6.6		Isometric knee flexion at 15°, 30°, and 45° knee position	∼130 ± 20	r: 0.62 (volume) *p* < 0.001
						Isokinetic knee flexion at 50°/s	∼110 ± 25	r: 0.74 (volume) *p* < 0.001
						350°/s	∼65 ± 15	r: 0.71 (volume) *p* < 0.001
Gadeberg et al. (1999)	18 healthy adults (12 male, 6 female), mean age = 41 (29–64) years	Plantar flexors	1607 ± 396	115 ± 25		Isokinetic plantar flexion at 60°/s	117 ± 26	r^2^: 0.69 (volume) *p* < 5 × 10^−5^
								r^2^: 0.46 (CSA) *p* < 5 × 10^−3^
		Dorsiflexors	363 ± 97	27 ± 6		Isokinetic dorsiflexion at 60°/s	32 ± 8 Nm	r^2^: 0.76 (volume) *p* < 5 × 10^−6^
								r^2^: 0.73 (CSA) *p* < 5 × 10^−5^
Hori et al. (2020)	30 healthy male, age = 21.9 ± 1.8 years	Quadriceps	2015.4 ± 288.5		43.8 ± 2.4	Isometric knee extension at 90° knee position	244.8 ± 49.8	r: 0.785 (volume) *p* < 0.001
								r: 0.790 (moment arm) *p* < 0.001
Trappe et al. (2001)	18 healthy adults (14 male, 4 female), age = 34.3 ± 2.4 years	Plantar flexors (calf muscles only)	642 ± 16	47.9 ± 1.3		Isometric plantar flexion at −10° DF	148.5 ± 40.2	r: 0.76 (between muscle size and peak isometric strength) *p* < 0.05
						0°	123.8 ± 33.6	
						10° PF ankle position	89.7 ± 24.2	

CSA, cross-sectional area; DF, dorsiflexion; MVC, maximum voluntary contraction; PCSA, physiological cross-sectional area; PF, plantar flexion; VL, vastus lateralis. Uncolored references show ankle joint function and related muscle-tendon unit parameters, whereas the gray-shaded ones are for the knee. All CSA, values represent the anatomical CSAs, of muscle groups determined based on MRI, images, whereas PCSA, values are estimated by combining the whole muscle group volume knowledge with target muscle-level information (i.e., fascicle length, pennation angle) obtained from the US., Values are reported as average ± standard deviation. In the studies testing the ankle joint, the neutral ankle angle is specified as 0°, whereas, in those testing knee torques, 0° represents full knee extension and the exceptions were edited to ensure consistency between the studies. Please note that the interpretability of the published values should be approached with caution due to a notable sex imbalance within the studies featured in the table, with higher representation of males than females. Coefficients for determinations, either r^2^ or r, are indicated as specified in the original papers. *p* values for each correlation are indicated and *p* < 0.05 was considered significant.

The studies included in Table 1 (Gadeberg et al., 1999; Trappe et al., 2001; Baxter and Piazza, 2014), examined the entire posterior compartment as well as the calf muscles (gastrocnemii and soleus) within the compartment (Trappe et al., 2001). These investigations have revealed that calf muscles contribute roughly half of the total volume (average ±standard deviation: 642 ± 16 cm^3^ as opposed to 1607 ± 396 cm^3^) and CSA (47.9 ± 1.3 cm^2^ as opposed to 115 ± 25 cm^2^) of the posterior compartment. A recent study conducted on 38 healthy males (age = 21.6 ± 1.4 years) using MRI reported very similar dimensions for calf muscles (volume: 755.2 ± 115.8 cm^3^ and CSA: 46.1 ± 6.6 cm^2^) (Suga et al., 2021). Furthermore, there exists a notable correlation (r = 0.592, *p* < 0.001) between muscle volume and passive joint stiffness (defined as the slope of the linear portion of the torque-angle curve). In an assessment of ankle ROM during both passive and active dorsiflexion, a larger plantar flexor volume was associated with reduced dorsiflexion flexibility in a healthy population (Suga et al., 2021). These findings underscore that higher muscle volume not only affects passive joint stiffness but also plays a significant role in limiting flexibility during both passive and active dorsiflexion movements, emphasizing the multifaceted impact of calf muscle composition on ankle joint function in healthy individuals.

At the knee joint, significant correlations have been reported between extensor torque and quadriceps muscle size (volume and CSA), and flexor torque and hamstrings volume for both isometric and isokinetic activation types (Blazevich et al., 2009; Evangelidis et al., 2016; Hori et al., 2020; Balshaw et al., 2021). Notably, volumes of quadriceps and hamstrings account for a significant portion of inter-individual differences in strength during isometric and concentric contractions in both extensors (isometric 71%, concentric 30%–31%) and flexors (isometric 38%, concentric 50%–55%) (Evangelidis et al., 2016). Specifically, with the maximum voluntary knee extension torque, the volume of the quadriceps femoris muscle group produced the highest r-value (r = 0.773) followed by the CSA and muscle thickness, such that qualitatively volume explained 60% of the variance in knee extension strength (Balshaw et al., 2021). In addition to the whole quadriceps volume measured, the volume of each constituent muscle (vastus lateralis, vastus intermedius, vastus medialis, and rectus femoris) in the quadriceps muscle group had been measured as well. Yet, the authors did not provide any statistical analysis regarding the one-to-one relationship of each muscle size with knee extension torque.

Muscle moment arms are responsible for transforming muscle force and linear displacement into joint angular movement (Maganaris, 2004b). It is widely accepted that muscle’s contribution to joint torque is proportional to its moment arm; the larger moment arms result in greater MTU movements and joint moments (Maganaris, 2004b; Kellis and Blazevich, 2022). At the ankle joint, the correlation between plantar flexion torque (measured both isometrically and isokinetically) and the triceps surae moment arm is at least as strong as those found between torque and muscle volume (providing even higher *p*-values when measured isokinetically), highlighting the significance of moment arm in determining strength (Baxter and Piazza, 2014).

Conversely, at the knee joint, the relationship between knee extensor torque and the moment arm is more nuanced. Blazevich et al. (2009) found a weak but significant correlation between the knee extension torque and the quadriceps moment arm only in isometric conditions, not in isokinetic knee extensions (Table 1). A recent study corroborated such a correlation for isometric activation (Hori et al., 2020). However, in a study performed in preadolescent boys (age = 10.7 ± 0.9 years), the quadriceps femoris volume and knee extensor moment arm were measured together with the knee extensor isometric and isokinetic torques (at slow and fast angular velocities) (Tottori et al., 2020), and both quadriceps volume (699.2 ± 158.4 cm^3^) and moment arm (34.3 ± 2.4 mm) were reported to be significantly correlated with all knee extensor torques measured in isometric and isokinetic activations (r = 0.513–0.804, *p* < 0.05 for volume, and r = 0.701–0.806, *p* ≤ 0.001 for moment arm).

Importantly, moment arm exhibits variability along the moving axis of the joint. However, bone size is not the sole parameter to affects the moment arm, such that only a few muscles have a significant correlation between their mean moment arm and joint angular position (Klein et al., 1996). For example, the moment arm for the Achilles and tibialis anterior tendons are longer during maximum voluntary contraction (MVC) compared with rest, and this is not only due to the change in joint positioning but also muscle thickening and stretching of collagenous connections mediating the action of the tendon (Maganaris, 2004b). These point out the importance of considering the moment arm variations based on activation type and intensity as well as the measurement method.

Moment arms can be measured *in vivo* using computed tomography and MRI (Németh and Ohlsén, 1985; Spoor and van Leeuwen, 1992; Wilson and Sheehan, 2009). Over the past 2 decades, biomechanical models have gained popularity in simulating movement, allowing for computing the moment arms and MTU lengths as well (Delp et al., 1990; Liu et al., 2006). In their study, using MRI images, Arnold et al. (2000) developed musculoskeletal (MSK) models and compared the hip and knee flexion moment arms of the medial hamstrings and psoas muscles calculated with these models with the experimental data. Based on the findings, MSK modeling provides an accurate and efficient means of estimating MTU lengths and moment arms *in vivo*. As the variations in MTU parameters (e.g., moment arm) would affect model predictions of muscle function during movement, the sensitivity of each specific parameter needs to be well-understood. A Monte-Carlo analysis revealed that changing the moment arm of a given muscle results in task-specific changes in the muscle’s force and its contribution not only to the joint torque it spans but also to the joint torques generated by adjacent and non-perturbed muscles (Ackland et al., 2012). For example, manipulating the moment arm of the iliopsoas leads to a greater change in the hip flexion torque generated by the rectus femoris than that generated by the iliopsoas. The functional roles of muscles during walking are highly sensitive to the changes in MTU properties (e.g., tendon slack length and optimal fiber length) and relatively less sensitive to moment arms (Ackland et al., 2012). Using MRI imaging, Hori et al. (2020) recently tested whether a longer (knee extensor) moment arm would be related to higher torque-producing capacity in healthy young men despite a given muscle size (quadriceps volume). There were significant correlations between the quadriceps femoris volume and knee extensor moment arm with the knee extensor isometric torque. The knee extensor moment arm correlated significantly also with the knee extensor torque-producing capacity. These findings suggest that a longer moment arm is a substantial factor in achieving higher torque production in the human knee extensors. The torque-producing capacity (i.e., the maximal isometric torque per muscle size) is, therefore, affected by both muscle size and moment arm.

### 2.2 Muscle size and moment arm vs. muscle force production

Simulation of human movement has become useful in predicting the force-generating capacity of lower limb muscles. Using a Hill-type model, examination of the muscles’ force and moment generation capacities at the ankle, knee, and hip joints is possible (Arnold et al., 2010). However, these modeling studies are based on several assumptions (Lenaerts et al., 2009; Scheys et al., 2011; Gerus et al., 2013; Valente et al., 2014), and their effects on model outcomes are non-negligible (Out et al., 1996). Recent sensitivity analysis by quantifying the impact of potential errors in MTU parameters (Carbone et al., 2016) showed which MTU parameters for which muscle should be estimated most accurately when a Hill-type MSK model is implemented. It appeared that during a simulated gait cycle, in addition to the size and length of the MTU parts of the tested muscles (including gastrocnemius, soleus, rectus femoris, vastus lateralis, tibialis anterior), other parameters such as tendon slack length, maximal isometric muscle force, and optimal fiber length have significant influences on the creation of a reliable subject-specific MSK model that satisfies the required accuracy of the specific application.

Electromyography- (EMG) and ultrasound (US) imaging-driven models are used to estimate *in vivo* muscle force based on joint torque information (Lloyd and Besier, 2003; Lichtwark and Wilson, 2006; de Oliveira and Menegaldo, 2010; Sartori et al., 2012; Pizzolato et al., 2015; Dick et al., 2016; Hoang et al., 2018; Veerkamp et al., 2019). Yet, as some parameters like the maximum voluntary contraction force vary widely among individuals, decreasing model errors is of great importance. A Hill-type EMG-driven model developed for ankle joint torque prediction showed that subject-specific muscle maximum force estimation using the US-obtained PCSA values of calf muscles significantly decreases the torque prediction errors compared to a not-used scenario (de Oliveira and Menegaldo, 2010). In a more general manner, any realistic modeling of joint moment-angle relationships needs to reflect the combined effects of PCSA, the moment arm, the pennation angle, force-length relations of muscle fibers, and the muscle-specific tension (Hoy et al., 1990; Out et al., 1996; van den Bogert et al., 1998; Maganaris, 2004a; O’Brien et al., 2010; Carbone et al., 2016; Meyer et al., 2017; Lieber, 2022).

The usage of MSK simulation tools such as AnyBody (Damsgaard et al., 2006) or OpenSim (Delp et al., 1990) and processing 3D motion analysis data based on an inverse dynamic approach is promising for estimating subject-specific muscle forces and muscle activity levels during movements (van der Krogt et al., 2012; Trinler et al., 2019). Good agreements of the model-computed muscle activity levels with measured EMG data have been reported during walking (van der Krogt et al., 2012; Alexander and Schwameder, 2016). In their study, van der Krogt et al. (2012) simulated the normal walking of healthy adolescents using OpenSim software and by progressively weakening all major muscle groups, one at a time and simultaneously, how much weakness could be tolerated before execution of normal gait became impossible has been evaluated: Normal walking is remarkably robust to the weakness of some muscles (e.g., hip and knee extensors) but sensitive to the weakness of others (e.g., plantar flexors, hip abductors, and hip flexors), suggesting that certain compensations arise as a result of weakening some target muscles. By clarifying which muscles are critical for maintaining normal gait and how much their weakening alters the mechanics of other muscles and related joints, this study offered important insights. Recently, Uhlrich et al. (2022) simulated knee contact forces based on EMG data, using OpenSim. Their objective was to identify and examine simple changes in muscle coordination during walking with the therapeutic goal of reducing joint loading. After a session of walking with biofeedback on plantar flexor muscle activation, tested healthy young adults were able to reduce their late-stance knee contact forces by altering the ratio of gastrocnemius-to-soleus muscle activation. The study quantified the impact of certain muscle groups (quadriceps, hamstrings, gastrocnemius) on knee contact forces, and suggested a gait pattern that avoids excessive use of gastrocnemius as a way of reducing knee joint loading. By understanding the individual contribution of muscles to joint loading and function, it may be possible to train individuals with certain conditions to adopt new and therapeutic coordination strategies.

It is clear that the more we know about individual muscle forces during specific movements and how they affect joint functions, the better we can prevent or ameliorate gait pathology observed in, for example, neuromuscular disorders. Although this is one of the main motivations for MSK modeling and these approaches are becoming more common in biomechanical research, they have not yet become established in routine clinical practice (Trinler et al., 2018; Killen et al., 2020; Fregly, 2021). One reason for that is substantial differences in some phases of the gait cycle between existing MSK models regarding muscle force estimations (Trinler et al., 2018). This is mainly because various assumptions and simplifications are being used in each model when calculating model parameters (Lin et al., 2012; Zelik and Honert, 2018). There are differences in anatomical definitions (e.g., pelvis definition to the ground, mass, and inertia of the segments) or the calculation of joint centers (e.g., through different scaling approaches, model marker placement) (Trinler et al., 2019). Different optimization techniques (i.e., static and enhanced static, and forward optimization models) are being implemented in each mathematical model to solve the redundancy problem (i.e., more muscles spanning a joint than degrees of freedom exist) (Trinler et al., 2018). Optimizations allow the minimization of specific cost functions being used, e.g., for resolving load-sharing problems (Praagman et al., 2006; Trinler et al., 2018). Encoding the desired task into a cost function, a mathematical definition of the nature of the solution being sought is provided in the model (Nikoo and Uchida, 2022). However, the simplifications made in the models neglect some parameters that are quite effective in reality, such as working with fewer muscles than the actual number spanning the joint being examined, or not taking into account the activation of the antagonists, which leads to a lower net joint moment compared to the agonist muscles alone (Reeves et al., 2003; Maganaris, 2004a), or the segmental interactions and transmission of force through these interactions between activated muscles (Yucesoy, 2010; Kaya et al., 2020).

Estimating muscle forces from joint torque aside, measuring them directly from the tendon of the targeted muscle would be an ideal solution (Komi et al., 1992; Yucesoy et al., 2010). Intraoperatively, it is possible to quantify the force production capacity of a muscle throughout the whole ROM. It requires fixing a buckle force transducer over the target muscle’s tendon for capturing the force values and placing stimulation electrodes on the skin over the muscle bellies for activating muscles. Using this method, the gracilis muscle force-knee joint angle characteristics have been demonstrated in healthy young adults (who were undergoing anterior cruciate ligament reconstruction surgery) (Yucesoy et al., 2010). The findings indicated substantial inter-individual variability and suggested that the typical subject anthropometrics (e.g., mass, mid-thigh perimeter, thigh length) cannot be used safely as predictors of the contribution of human muscles to joint moments. Similarly, the analysis of how stress scales with force yielded a huge inter-subject variability for peak gracilis tendon stress and indicated no fully consistent scaling of tendon size (CSA) and force. Recently a similar intraoperative approach was applied to test also the gracilis muscle in patients with brachial plexus injury (who were planned for gracilis muscle transfer) and suggested this technique as an essential tool for understanding *in vivo* muscle function as well as developing computational muscle models (Persad et al., 2022). Apart from the scientific perspectives that the direct measurement of the biomechanical properties of the human muscle (i.e., muscle force-joint angle) brings, 1) muscle force data would improve and validate the muscle and MSK models for better predictions, and 2) the implementation of this intraoperative data collection to the surgical routine would provide immediate feedback to the surgeons during, e.g., tendon lengthening or transfer operations.

## 3 The contribution of the muscle-tendon unit to joint function in patients with cerebral palsy

CP is a neurological disorder stemming from brain lesions affecting body coordination, profoundly impairs the proper functioning of the musculoskeletal system. This impairment leads to enduring disabilities in posture and movement. The persistence of exaggerated stretch reflexes results in stretch resistance in muscles which can further lead to contracture formation (Botte et al., 1988; Gracies et al., 2010). Patients commonly experience elevated joint and muscle stiffness (Botte et al., 1988; Gracies, 2005; Bénard et al., 2010; Smith et al., 2011; de Bruin et al., 2014) and restricted ROM (Elder et al., 2003; Fergusson et al., 2007; Alhusaini et al., 2010). Despite the functional limitations led by these pathologies being obvious, their biomechanical sources remain less clear.

*Muscle Growth and Weakness:* Impairments in overall muscle growth are routine in patients and may have appeared in the form of muscle volume reduction, thinning, PCSA decreasing, and compromised muscle and tendon length, among other factors (Table 2) (Barrett and Lichtwark, 2010; Kaya Keles and Ates, 2022). Muscular deformity is often associated with spasticity, which describes repetitive and uncontrolled muscle contractions as a consequence of increased resistance to stretch. However, its development could be related to an impairment of muscle growth and altered adaptation (Gough and Shortland, 2012). A previous study by Handsfield et al. (2014) utilizing fast-acquisition MRI to measure volumes and lengths of 35 major lower limb muscles in healthy young adults showed that these muscles proportionally scale with bone geometry and subject size parameters (such as mass and height) to achieve comparable functionality across individuals. Even though a linear relationship was observed between body mass and total lower limb muscle volume for patients with CP, similar to healthy individuals, this relationship exhibited a 40.6% shallower slope for CP compared to the control group (Noble et al., 2017). Regarding the muscle size alterations, in children around 10 years old, MRI scans of 6 lower limb muscles demonstrated significantly smaller normalized volumes (i.e., for the hamstrings and the quadriceps by 26% and 22%, respectively) and shorter normalized muscle lengths (e.g., for gastrocnemius 49% ± 3.9% and 58% ± 5.8% for the patient and control groups, respectively) in the patients than those in the control group (Oberhofer et al., 2010). While muscle volumes correlated with body mass in healthy subjects, this relationship held true for only 5 out of 6 muscles in patients with CP. Regression analyses between segment lengths and muscle lengths yielded variable results for each muscle and segment group in both cohorts. For instance, there was no significant correlation between soleus lengths and shank lengths in the patient group (r^2^ = 0.375), while such a correlation existed in the control group (r^2^ = 0.680) (Oberhofer et al., 2010). In children aged 2–5 years, the medial gastrocnemius muscle volume was 22% smaller in the group with spastic CP compared to the typically developing (TD) children (25 ± 2 mL and 33 ± 2 mL, respectively) (Barber et al., 2011b). Deficits in muscle volume are observed in the spastic muscles as early as the age of 15 months (Herskind et al., 2016). Moreover, the increase in medial gastrocnemius volume with age is less in the CP group, compared to that in TD (r^2^ = 0.82 and 0.37, for the TD and CP groups, respectively). Such volume deficits are correlated with the severity of the disease: The most severely affected children with CP showed a larger reduction in muscle growth compared to the age-matched medial gastrocnemius volume (Gross Motor Function Classification System (GMFCS) level I: 7.9 ± 7.4 mL; GMFCS II: 12.84 ± 10.4 mL; GMFCS III: 19.1 ± 5.0 mL), while no significant difference was reported for the increase in bone length with age between the CP and TD groups (Herskind et al., 2016). These indicate that pathology-related problems rather than growing- or age-related factors dominate muscular adaptation in individuals with CP at later ages.

**TABLE 2:** Selected studies investigating the joint function together with the muscle-tendon unit parameters in patients with CP.

Reference	Study population	Muscle tested	Group	Muscle-tendon unit parameters				Joint function			
Barber et al. (2011a)	CP (*n* = 9 (male/female: 6/3); 17 ± 2 years; GMFCS: I) TD (*n* = 10 (male/female: 5/5); 18 ± 2 years)	Medial gastrocnemius		*Volume (mL)*	*PCSA (cm* ^*2*^ *)*	*Fascicle length change (mm)*		*Direction of movement*	*Max dorsiflexion angle (˚)*	*Ankle ROM (˚)*	*Peak passive ankle torque (Nm)*
			TD	**223 ± 21**	**53 ± 5**	**9 ± 1**		Plantar flexion	**21 ± 1**	**21.7 ± 1**	11.4 ± 1
			CP	134 ± 20*	34 ± 3*	4 ± 1*			6 ± 1*	14.9 ± 1*	10.1 ± 1
Haberfehlner et al. (2016)	CP (*n* = 9 (male/female: 4/5); 14.1 ± 2.7 years; GMFCS: II-III) TD (*n* = 9 (male/female: 4/5); 14.1 ± 3.2 years)	Semitendinosus		*Volume (cm* ^*3*^ *)*	*Muscle length (% of femur length)*	*MTU length (% of femur length)*	*Fascicle length (% of femur length)*	*Activation type*	*Max knee extension angle* ^*(a)*^ *(˚)*	*Knee ROM* ^*(a)*^ *(˚)*	*Peak knee moment (Nm)*
			TD	**96.0 ± 22.3**	81.6 ± 8.3	123.1 ± 6.9	**48.9 ± 4.4**	Knee flexion	**31.5 ± 6.8**	**41.0 ± 8.1**	5.8 ± 1.3
			CP	36.6 ± 17.8*	74.6 ± 6.6	121.2 ± 5.7	38.2 ± 5.1*		**54.1 ± 11.8**	29.4 ± 5.4*	5.2 ± 1.9
Hanssen et al. (2021)	CP (*n* = 53 (male/female: 31/22); 8.2 ± 4.1 years; GMFCS: I-III) TD (*n* = 31 (male/female: 15/16); 9.7 ± 2.9 years)			*Volume (mL)*	*Volume normalized to body mass (mL/kg)*	*Muscle length (mm)*	*Muscle length normalized to body height (mm/cm)*	*Activation type (isometric)*	*Peak torque (Nm)*		
		Rectus femoris	TD	**96.0 ± 32.0**	**3.17 ± 0.61**	**256.8 ± 47.1**	1.84 ± 0.23	Knee extension	**31.0 ± 35.5**		
			CP	64.9 ± 33.2*	2.30 ± 0.63*	224.5 ± 47.5*	1.75 ± 0.16		13.3 ± 18.4*		
		Semitendinosus	TD	**68.8 ± 31.3**	**2.24 ± 0.75**	**244.1 ± 50.8**	**1.81 ± 0.28**	Knee flexion	**22.5 ± 11.9**		
			CP	48.8 ± 30.6*	1.80 ± 0.36*	221.0 ± 46.7*	1.68 ± 0.21*		6.7 ± 10.4*		
		Medial gastrocnemius	TD	**68.5 ± 29.1**	**2.44 ± 0.41**	**185.5 ± 39.6**	**1.35 ± 0.15**	Plantar flexion	**12.5 ± 8.3**		
			CP	40.0 ± 26.4*	1.49 ± 0.76*	146.1 ± 39.9*	1.19 ± 0.24*		7.0 ± 5.2*		
		Tibialis anterior	TD	**48.6 ± 28.7**	**1.60 ± 0.49**	**242.9 ± 58.0**	**1.74 ± 0.23**	Dorsi-flexion	**9.9 ± 6.0**		
			CP	25.5 ± 12.6*	0.90 ± 0.25*	198.2 ± 43.7*	1.58 ± 0.20*		2.3 ± 2.0*		
Kalkman et al. (2018)	CP (*n* = 15 (male/female: 10/5); 11.4 ± 3 years; GMFCS: I-II) TD (*n* = 16 (male/female: 7/9); 10.3 ± 3 years)	Medial gastrocnemius		*Muscle length change (mm)*	*Tendon length change (mm)*	*Fascicle length change (mm)*		*Direction of movement*	*Ankle ROM (˚)*	*Passive ankle torque* ^*(b)*^ *(Nm)*	
			TD	**26.5 ± 7.0**	16.8 ± 6.7	**26.0 ± 4.3**		Plantar flexion	**60.6 ± 11.0**	**0.49 ± 0.94**	
			CP	18.2 ± 5.4*	20.7 ± 8.1	15.9 ± 6.2*			**48.0 ± 12.8**	**2.34 ± 1.77**	

CP, cerebral palsy; GMFCS, gross motor function classification system; PCSA, physiological cross-sectional area; ROM, range of motion; TD, typically developing. Uncolored references show ankle joint function and related muscle-tendon unit parameters, whereas the gray-shaded ones are for the knee. Values are reported as average ± standard deviation. In the studies testing the ankle joint, the neutral ankle angle is specified as 0°, whereas in those testing the knee joint, 0° represents full extension. Significant group differences (*p* < 0.05) are indicated in bold font and * indicates *p* < 0.01, if reported in the original papers. ^(a)^ The subjects were positioned with the hip of their measured leg at 70° flexion and the knee joint function parameter was assessed not for the entire ROM, but instead between knee angles corresponding to a knee flexion moment production of 0 and 4 Nm. ^(b)^ Measurements were done at −5° (dorsiflexion) as that was the most dorsiflexed position that all subjects in both groups could achieve.

One common clinical symptom in children with CP is muscle weakness (Elder et al., 2003; Mockford and Caulton, 2010), characterized by the impairment of the ability to generate or maintain the required level of force (Edwards, 1978). This weakness primarily has a neural basis due to CP’s neurological origin. However, in addition to neural factors, a muscle’s strength is also influenced by its intrinsic capacity (Elder et al., 2003; Mockford and Caulton, 2010). The failure of affected muscles to grow raises a very essential question: how do deficits in muscle size contribute to muscle weakness? Understanding the individual contributions of muscle contractures to resulting joint pathologies is vital, especially since the affected muscle is often directly targeted in treatments, such as local injection of botulinum toxin or surgical lengthening. While widely used clinical techniques, such as clinical evaluations of joint ROM, popliteal angle, and gait analysis, are effective at estimating joint restrictions, they do not directly indicate the status of muscles and their levels of deformation, which would greatly improve the therapy.

For example, in their study on the limited passive ankle ROM in the paretic limbs of patients with CP compared to those of TD individuals, Malaiya et al. (2007) sought a relationship between this limitation and the size of the medial gastrocnemius muscle belly. Assessment of US images indicated that the medial gastrocnemius in the paretic limbs of the patients has a smaller volume and shorter belly length, with no difference in normalized fascicle lengths between groups. These findings led the authors to conclude that the lack of longitudinal growth in the paretic muscles is not due to reduced fascicle length, but rather due to a lack of cross-sectional growth. No direct relationship between muscle length and ankle angle was found though. Presently, there is consistent evidence that muscle belly length is shorter in the paretic muscles of the patients compared with their TD counterparts in absolute and relative terms (Barrett and Lichtwark, 2010). However, the findings on fascicle length comparisons between the individuals with CP and their TD counterparts are inconsistent in the literature (Barrett and Lichtwark, 2010). Some studies reported no difference (Shortland et al., 2002; Malaiya et al., 2007; Barber et al., 2011b; Mathewson et al., 2015; Herskind et al., 2016) while others pointed out significantly shorter fascicle lengths in the pathological muscles of the patients (Lieber et al., 2003; Mohagheghi et al., 2007; 2008). In cases where no difference in fascicle length is reported, one suggestion is that the muscle volume deficits in the CP may result from smaller fiber diameters or fewer muscle fibers within the muscles (Herskind et al., 2016). The origins of these differences remain unclear, whether a reduction in muscle fiber diameter and/or the number of fibers is responsible. Nevertheless, the most consistent finding is that muscle volume, thickness, or PCSA are smaller in patients compared to their TD peers (Barrett and Lichtwark, 2010).

### 3.1 Muscles crossing the ankle joint

*Passive State:* Compared to the TD group, individuals with CP exhibit several key differences in their passive ankle characteristics. They have a notably narrower maximum dorsiflexion angle at rest (i.e., an indicator of a limited passive ankle ROM) and greater ankle stiffness (i.e., the slope of the ankle torque-angle curve across the measured ankle angle range) (Table 2) (Barber et al., 2011a). Additionally, the medial gastrocnemius muscles are significantly smaller in the CP group in terms of both muscle volume and PCSA, and their mean fascicle length range (i.e., the difference between its length at maximum dorsiflexion and its slack length) is lower (Barber et al., 2011a). Moreover, the CP group exhibits a steeper slope of the ankle torque *versus* the medial gastrocnemius fascicle length curve, indicating increased muscle stiffness in CP. This suggests that the reduced passive ankle ROM and increased ankle joint stiffness due to CP are related to the inability of medial gastrocnemius muscle fascicles to elongate adequately under passive force. These findings confirm the expectation that deficits in muscle size contribute to the observed joint pathology. In a study by Kalkman et al. (2018), the contribution of both muscle and tendon to joint limitation in children with CP was investigated. It was found that ankle ROM is significantly smaller towards dorsiflexion in CP, and at the most dorsiflexed position that all participants could achieve, joint torques were significantly larger than those in TD. Over the ROM achieved by all participants, when the ankle joint angle was passively changed in dorsiflexion direction, the muscle lengthens by 63%, and the tendon lengthens only 37% in TD individuals. In contrast, in patients with CP, both the muscle and tendon lengthened equally, indicating greater stiffness of the muscle relative to the tendon, and elevated relative contribution of the tendon to MTU lengthening.

Several studies using imaging techniques (Kwon et al., 2012; Brandenburg et al., 2016; Lallemant-Dudek et al., 2021) or performing histological analysis on muscle biopsies and quantifying changes in intramuscular connective tissues (Booth et al., 2001; Smith et al., 2011), have shown greater stiffness for various muscles in patients with CP compared to healthy individuals, linking such increased stiffness to impaired function (Vaz et al., 2006). In children with CP, stretching calf muscles passively revealed that the lengthening of both muscle and tendon portions of the medial gastrocnemius is directly related to the ankle ROM, a not found in TD children (Kalkman et al., 2018). Therefore, the alterations observed in CP (e.g., elevated muscular stiffness, deficits in muscle PCSA, and MTU length) contribute to the pathological function (Kalkman et al., 2018). This indicates that altered material properties of both muscles and tendons need to be taken into consideration when planning treatment strategies to improve function in patients.

*Active State:* To identify which muscle-tendon structures are responsible for the pathological active ROM, Barber et al. (2012) analyzed the ankle joint active torque production in relation to the medial gastrocnemius muscle architecture and the Achilles tendon length. They discovered that the absolute active ankle plantar flexion torque was significantly lower (by 33%) for the CP group (27.1 ± 4.8 Nm) compared to the TD group (60.8 ± 4.5 Nm). However, when normalized to PCSA, there was no significant difference in active plantar flexion torque between the groups (0.9 ± 0.3 Nm/cm^2^ and 1.1 ± 0.3 Nm/cm^2^, for CP and TD groups, respectively). The CP group had a significantly lower medial gastrocnemius PCSA (by 37%) (Barber et al., 2011a). These suggest that PCSA plays a major role in muscle weakness and, consequently, restricted active ankle ROM. No significant differences were found for the active medial gastrocnemius fascicle length between the two groups, while the Achilles tendon slack length was significantly longer in the CP group, though there was no difference in its stiffness when computed in absolute or relative terms. This longer Achilles tendon slack length may allow enhanced storage and recovery of elastic energy, e.g., during walking. This may be a favorable adaptation in CP which partially compensates for the decreased force production capacity of the contractured, i.e., smaller, and shorter muscles, which are unable to span an appropriate ROM. Furthermore, during isometric plantar flexion MVC, there is significantly higher co-contraction of the antagonist tibialis anterior in the CP group (Elder et al., 2003; Stackhouse et al., 2005). Combining all, it is reasonable to conclude that the decreased ankle joint strength during maximal contractions in children with mild CP resulted from smaller medial gastrocnemius muscles and increased co-contraction of the tibialis anterior. On the other hand, Hanssen et al. (2021) found a disproportional relationship between muscle size and weakness: When the volumes of the medial gastrocnemius and tibialis anterior muscles were normalized to body mass, and muscle length to body height, both were significantly lower in the CP group compared to TD children. Normalized maximum joint torque was also lower in CP for both plantar flexion and dorsiflexion. The ratio of joint torque over muscle size was significantly lower in the CP group only for dorsiflexion. For plantar flexion, observed differences were not statistically significant. They reported significant correlations between medial gastrocnemius muscle volume and maximal plantar flexion torque as well as tibialis anterior muscle volume and maximal dorsiflexion torque for both groups. However, the regression coefficients (i.e., slopes of the relations) tended to be lower in the CP indicating that only part of the muscle weakness can be attributed to smaller muscle volumes. This investigation confirms the disproportional decreases in muscle size and muscle strength around the ankle joint in children with CP compared to TD children and highlights the large variability in the contribution of muscle size to muscle weakness.

### 3.2 Muscles crossing the knee joint

*Passive State:* The comparisons of the semitendinosus size and knee moment-angle characteristics between CP and TD groups demonstrated significant differences. In children with CP, the knee moment-angle curve shifts towards more flexed knee angle positions, indicating limited knee ROM, and it has a steeper slope, signifying higher stiffness (Haberfehlner et al., 2016). Interestingly, MTU lengths normalized to the femur lengths were similar in both groups (Table 2), indicating no significant difference in bone length between groups. However, the CP group exhibits smaller muscle volume, PCSA, and muscle fascicle lengths normalized to femur lengths compared to TD children. This relationship between smaller muscle sizes and lower force generation suggests that most children with CP have weaker semitendinosus muscles compared to their TD counterparts. Multiple regression analyses, assessing the relationship between knee moment-angle characteristics and muscle architecture, reveal that a steeper slope of the knee moment-angle curve in the semitendinosus muscles of the patients is mainly determined by shorter fascicle lengths.

*Active State:* The rectus femoris and semitendinosus volumes normalized to body mass were shown to be significantly smaller in the CP (Hanssen et al., 2021) however, only the muscle length normalized to the body height of semitendinosus was smaller (Table 2). Notably, normalized maximum joint torque values for both knee extension and flexion MVCs were significantly lower in CP. The ratio of joint torque over muscle size was also smaller in the CP group for both flexion and extension of the knee. The correlation coefficients between rectus femoris volume and maximal knee extension torque were similar for TD and CP, with the slope of this relation tending to be lower in the CP indicating that only part of the muscle weakness can be attributed to smaller muscle volumes. For the semitendinosus muscle, no difference in the slope of the muscle volume-maximal knee flexion torque curve was found between groups. The disproportional decreases in muscle size and muscle strength around the knee joint of children with CP in comparison to TD children have been shown though, substantial heterogeneity was observed in the proportion of muscle weakness that was attributed to deficits in muscle size both between joint torques and between patients, point the effectiveness of other mechanisms underlying muscle weakness. The associations of maximal joint torque with growth-related parameters (e.g., age, weight, and height) appeared strongest in the TD group, whereas for the CP group, these were accompanied by associations with selective motor control and GMFCS levels.

Similarly, a study in which the muscle size–strength relationships of the knee flexors and extensors were assessed in children with CP (*n* = 18, age = 7 year 5 month ± 1 year 7 month) and in relation to TD children (*n* = 19, age = 7 year 6 month ± 1 year 9 month) revealed smaller flexor (volume: 127.3 ± 36.5 cm^3^, and CSA; 6.6 ± 1.8 cm^2^) and extensor muscle groups (volume: 287.9 ± 76.1 cm^3^, CSA: 12.9 ± 4.0 cm^2^) in the patients in comparison to healthy peers (volume: 178.7 ± 53.7 cm^3^ and 382.2 ± 101.4 cm^3^ and CSA:14.2 ± 2.8 cm^2^ and 25.3 ± 4.9 cm^2^ for flexors and extensors, respectively) (Reid et al., 2015). These reported values represent a reduction of 29% and 25% of muscle volume and 54% and 49% of CSA in CP for flexors and extensors, respectively. Additional to the changes in muscle sizes, children with CP demonstrated significant weakness across all measures of strength for both the knee flexors and extensors as well. Children with CP were weaker than their TD peers across all torque variables: Isometric and isokinetic peak torques normalized by body mass were 65.4 ± 28.8 and 67.2 ± 28.8 Nm/kg for flexors and 186.8 ± 43.0 and 135.2 ± 38.9 Nm/kg for extensors in the CP group, whereas in TD group these values were equaling to 91.8 ± 24.0 and 87.7 ± 21.4 Nm/kg for flexors and 250.8 ± 65.6 and 168.1 ± 41.9 Nm/kg for extensors, respectively. These represent a reduced peak torque output both isometrically and isokinetically on average by 29% and 23% for flexors and 26% and 20% for extensors, compared to TD peers. For flexors, volume was significantly correlated both with the isometric (r = 0.54 and 0.84, for CP and TD groups, respectively) and isokinetic (r = 0.59 and 0.75, for CP and TD groups, respectively) peak torques. For extensors, volume was significantly correlated with the isometric peak torque only for TD (r = 0.77) but not CP and isokinetic (r = 0.51 and 0.67, for CP and TD groups, respectively) peak torques for both groups. These comprehensive correlation analyses concluded that children with CP have smaller, weaker knee flexor and extensor muscles than their TD peers. Yet, unlike their TD peers, muscle size does not necessarily correlate to muscle strength, and thus, muscle volume may only partially explain their decreased torque capacity.

### 3.3 Force characteristics of contractured muscles

**FIGURE 1:**
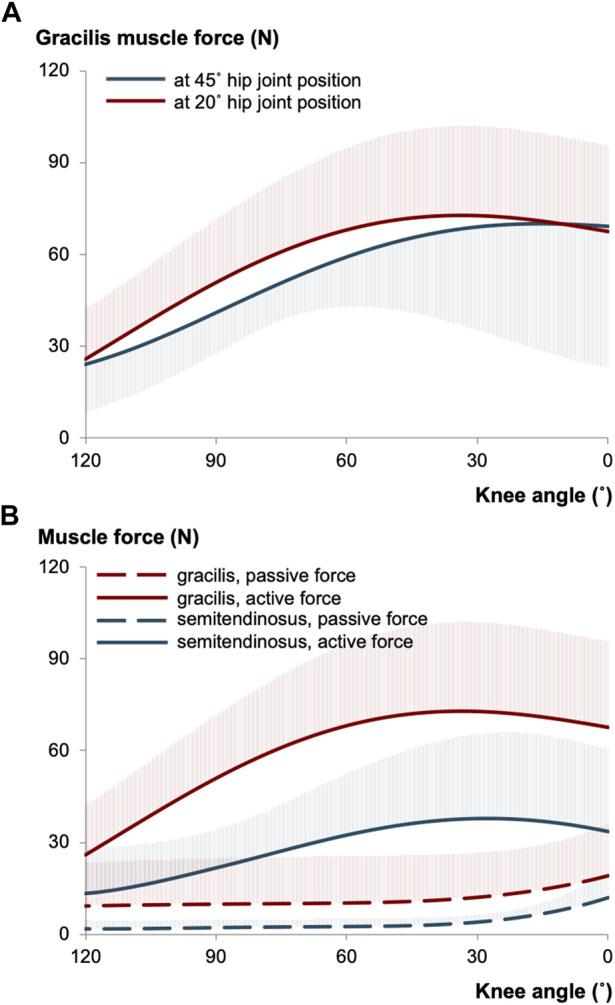
**(A)** The mean of gracilis muscle forces measured intraoperatively at different hip joint positions per knee angle in patients with CP (illustrated using the data collected previously (Kaya et al., 2019)). **(B)** The mean of gracilis and semitendinosus muscle forces of intraoperatively tested CP patients (illustrated based on (Kaya et al., 2019; Kaya et al., 2020)). The hip joint was fixed at 20° of flexion, whereas the knee joint position was altered during testing. The shaded areas in each curve show the standard deviations for one direction only and apply equally to the other direction.

Over the last decade, several intraoperative measurements were conducted to elucidate the force-joint angle characteristics of knee flexor muscles (e.g., Ates et al., 2013; 2016; Yucesoy et al., 2017). The primary goal has been to understand the contributions of individual muscles to the pathological joint function observed in patients with CP. For instance, research on gracilis muscle has revealed intriguing findings. Gracilis exhibits its minimum force in the most flexed knee joint position, show an increase in response to added knee extension, and reach a maximum before decreasing (Ates et al., 2013; Kaya et al., 2019). These findings align qualitatively with the force-joint angle curves of gracilis of healthy individuals (Yucesoy et al., 2010). Reasonably, the mechanical characteristics of the gracilis is expected to be influenced by its MTU length. As the gracilis also functions as a hip flexor, changes in hip joint positions impose length changes at its proximal end, which subsequently affect the distal end and changes its force-knee joint angle characteristics (Figure 1A): Gracilis muscle active forces were found to be around 13% higher when the hip joint is extended (at 20° flexion) compared to when it is relatively flexed (at 45° flexion) (Kaya et al., 2019). However, when the force-joint angle data are collected under almost identical conditions but from different patient groups with the same diagnosis (Ates et al., 2016; Ates et al., 2018b; Kaya et al., 2018), establishing a clear relationship between MTU length and force becomes challenging due individual differences. Moreover, the presence of high standard deviation values in mean force curves indicates substantial inter-individual variance.

Patients with CP display restricted knee ROM even in situations where there is no muscle activation involved (Farmer and James, 2001; Fergusson et al., 2007). This suggest that the pathological joint condition might be associated with non-contractile structures ascribed to stiff connective tissues such as the joint capsule or ligaments and/or high passive resistance of pathological muscles (Shortland et al., 2002; Sanger et al., 2003). The former often is detected in pre-operative clinical examinations and may prohibit full knee extension in a resting state (Ates et al., 2014). As mentioned above, the altered passive knee moment-angle characteristics of patients’ semitendinosus compared to those of TD children, indicate elevated connective tissue stiffness in CP (Haberfehlner et al., 2016), and muscle biopsy analyses support this finding by showing greater passive stiffness for patients’ hamstring fascicles (Smith et al., 2011).

Recent efforts have included direct quantification of muscle forces in a passive condition, representing the resting condition without any muscular activation. Figure 1B presents the passive and active forces of gracilis and semitendinosus muscles for the same patient group (Kaya et al., 2019; Kaya et al., 2020). Importantly, this patient group demonstrated a higher force production capacity of gracilis compared to that of semitendinosus, suggesting a potentially greater contribution of gracilis muscle to knee joint pathology. However, it is necessary to emphasize the word “capacity” here. The forces measured intraoperatively do not directly correspond to those acting on the joint during activities like walking. Instead, they quantify the maximum force that can be produced at the respective joint position (and corresponding MTU length). This information is particularly valuable for understanding other daily activities such as stair climbing, sit-to-stand, running, and jumping. Nevertheless, it is important to note that intraoperative force data primarily captures forces in isometric conditions, which do not fully represent most of the dynamic loadings. Nonetheless, once the force data of a specific muscle is obtained, the torque generated by that muscle at the relevant joint can be calculated using moment arm information.

Direct measurements of muscle forces during surgery have allowed researchers to collect data from selectively activated target muscles in various patient groups with CP across the full ROM of their knee (towards the 120° knee flexion to its full extension of 0°; the hip angle was kept at 0°) and the maximum force production capacities of the gracilis, semitendinosus, and semimembranosus muscles were reported as 41.59 ± 41.76 N (Ates et al., 2013), 87.6 ± 30.5–112.4 ± 54.3 N (Ates et al., 2016; Ates et al., 2018b), and 81.0 ± 54.7 N (Yucesoy et al., 2017), respectively. If we compare the force-knee joint angle characteristics of these three knee flexor muscles, we can better interpret their overall mechanical contributions on a limited knee ROM. In CP, contractured muscles are considered to adapt to shorter lengths. The reduced number of sarcomeres in series within a muscle fiber restricts its excursion (Tabary et al., 1972). Semimembranosus having much shorter fibers (fiber length: 6.90 ± 1.83 cm) than semitendinosus (19.30 ± 4.12 cm) and gracilis (22.78 ± 4.38 cm) (Ward et al., 2009) may indicate that contracture formation is more pronounced for semimembranosus, and this would reduce its excursion substantially (Yucesoy et al., 2017). The shape differences in force-angle curves observed might explain alterations in knee ROM: The gracilis has the greatest length range of force production capacity followed by the semitendinosus, which is ascribable to non-zero forces of gracilis during the entire excursion and its ascending portion of the knee joint angle-muscle force curve is being wider than that of the semimembranosus (Ates et al., 2013; 2016; Yucesoy et al., 2017). One more notable difference is that they reach their greatest force-generating capacities at different knee joint angles such that gracilis shows the greatest capacity reaching approximately 75% of its peak force by 90° of knee angle, whereas semitendinosus reaches about a third of its peak force and this is only about a 10th for semimembranosus by the same knee angle (Yucesoy et al., 2017).

Current knowledge of the relationship between individual muscle forces and their size (e.g., length) is limited. EMG-driven models have been employed to estimate muscle-level forces in children with CP, but these models may not fully account for motor control issues and muscle weakness associated with CP. Nevertheless, MSK models hold promise for clinical applications, including gaining insights into the underlying pathology, planning rehabilitation, and simulating post-operative scenarios (Veerkamp et al., 2019; Killen et al., 2020). For example, a model based on stretch data and EMG information for hamstring muscles in patients with CP and age-matched controls (van der Krogt et al., 2016) showed that hamstring muscles are stiffer in CP and vary between individuals, highlighting the need for personalized model parameters (van der Krogt et al., 2016). In dynamic tasks like gait, modeling the altered muscle properties associated with CP requires capturing patient-specific features, rather than using scaled generic parameters. Therefore, combining 3D motion capture data with EMG information is necessary to develop an MSK model. Recent studies have developed a simulation-based framework using highly personalized patient-specific models, including musculoskeletal geometry, muscle parameters, and motor control for selecting the most promising treatment option for individual patients based solely on pre-operative data (Pitto et al., 2019). Such a framework highlighted the importance of calibrated EMG-assisted modeling when estimating musculotendon forces in children with CP. However, conducting gait analysis and collecting extensive EMG recordings in children with CP is challenging and time-consuming. To address this, a muscle synergy extrapolation method was proposed to accurately estimate muscle activation patterns in individuals with CP collecting limited EMG data from only a few muscles of patients with CP and using available data from TD children (Rabbi et al., 2022).

In current research, efforts are focused on improving the prediction of muscle activation patterns in children with CP during clinical settings, emphasizing the need for a substantial experimental database for accurate analysis. Modeling has helped understand the relationship between muscle lengths and their force capacities, which have been directly measured intraoperatively. MSK modeling of the gait of patients with CP who were tested intraoperatively revealed that the semitendinosus and gracilis muscles were shorter compared to reference values (by 14% and 15%, respectively) (Kaya et al., 2020; Kaya and Yucesoy, 2020). However, the model does not distinguish the muscle belly and tendon lengths, it provides the total MTU length. Therefore, it cannot isolate shorter muscle length. To address this gap, patient-specific MTU lengths were then calculated using intraoperatively tested joint positions, and muscle force-length characteristics were derived by combining the intraoperatively obtained muscle force data with modeled MTU lengths for patients with CP (Kaya et al., 2020; Kaya and Yucesoy, 2020). Passive forces were increasing at longer MTU lengths, while active forces varied in a patient-specific manner (for details see the table in the Supplementary Material). Importantly, the absence of a strong correlation between shorter MTU length and higher force production found in patients with CP implies the need to consider other parameters, such as muscle force-velocity relation, intermuscular mechanical interactions (which provides an additional fascial pathway for transmission of force), rather than muscle length alone, to better understand the active mechanical behavior of muscles.

## 4 Discussion

### 4.1 Predictors of joint function

Numerous studies have demonstrated moderate to strong correlations between muscle size, moment arm, and joint function, all of which we review in this paper. In this section, we elaborate on the influence of size-related parameters of MTUs on a muscle’s force- and torque-generating capacity, drawing from an extensive body of literature.

While the quest to identify the ultimate predictor of joint moment, commonly referred to as muscle strength, has been ongoing, it's worth noting that no singular factor has emerged as the definitive predictor (Blazevich et al., 2009). Nevertheless, substantial evidence suggests that muscle size (characterized by metrics such as muscle volume, CSA, length, and PCSA) plays a critical and determinant role on its strength during different isometric and slow or fast isokinetic contractions in healthy and young populations. To provide context, consider the following torque values for healthy adults: The maximum ankle torques at a neutral position and knee at 90° flexion could produce are in the range of 104.2 ± 18.5–176.8 ± 21.8 Nm for plantar flexion (Trappe et al., 2001; Menegaldo and de Oliveira, 2009; Baxter and Piazza, 2014; Konrad et al., 2020) and 28.3 ± 8.0–49.9 ± 3.9 Nm for dorsiflexion (Geboers et al., 2000; Ruiz-Muñoz et al., 2017; Ates et al., 2018a), and 55.5 ± 14.1–72.0 ± 25.1 Nm for knee flexion (Ogborn et al., 2021; Nishida et al., 2022) and 177.3 ± 48.4–244.8 ± 49.8 Nm for knee extension (Blazevich et al., 2009; Dotan et al., 2013; Hori et al., 2020). It is important to note that, in contrast to the relatively balanced representation of males and females in ankle and knee flexion studies, a substantial majority of the data for knee extension (59 male/10 female) and ankle plantar flexion (70 male/4 female) was derived from male participants. Consequently, it is essential to be cautious when interpreting these findings, and future research should aim for a more equitable sex distribution to ensure data that is more representative of adult study cohorts.

While individual muscle sizes for healthy adults are less frequently reported, volumes for key muscle groups like calf muscles (642.0 ± 16.0–777.2 ± 103.7 cm^3^) (Trappe et al., 2001; Miyake et al., 2021; Suga et al., 2021), tibialis anterior (e.g., 131.8 ± 18.0 cm^3^) (Esformes et al., 2002), knee extensors (e.g., vastus lateralis occupying the biggest volume with 686.0 ± 254.2 cm^3^, compared to the vastus intermedius, vastus medialis, and rectus femoris (556.9 ± 200.6, 466.1 ± 153.2, and 259.7 ± 86.1 cm^3^, respectively), in a quadriceps muscle group with 1968.8 ± 675.2 cm^3^ total volume), and knee flexors (e.g., semimembranosus occupying the biggest volume with 234.1 ± 67.5 cm^3^, compared to the biceps femoris short head and long head, and semitendinosus (102.4 ± 41.9, 199.6 ± 65.2, and 203.1 ± 74.2 cm^3^, respectively), in a hamstrings muscle group with 739.2 ± 225.9 cm^3^ volume) (Kulas et al., 2018) are available in the literature.

Interestingly, the majority of literature tends to provide size-related information at the muscle group level rather than for individual muscles (Gadeberg et al., 1999; Baxter and Piazza, 2014; Hori et al., 2020). One notable exception is the work by Blazevich et al. (2009), which compared the relationship of the maximal knee extensor moment with the PCSA of the vastus lateralis muscle alone, in addition to with quadriceps muscles’ whole anatomical CSA. It was found that knee extensor moment produced at isometric and also at isokinetic conditions was significantly and positively correlated with both the CSA of quadriceps and the PCSA of vastus lateralis alone, although the correlations of quadriceps CSA with the joint function were stronger for all knee extension movements. This observation prompts the question of whether analyzing muscle group size is superior to individual muscle analysis in predicting joint function. Surprisingly, no statistically significant differences were found for the extensor moment prediction capability of muscle volume, or anatomical CSA of quadriceps and vastus lateralis PCSA indicating that analyzing the size of a muscle group rather than an individual muscle alone may not lead to a better statistical prediction of joint function. On the contrary, ranking the involvement of each muscle crossing the related joint would be more helpful in understanding the joint function. The notion of load sharing among quadriceps muscles during knee extension further emphasizes the importance of individual muscle analysis. Studies (e.g., Zhang et al., 2003) have shown that during isometric MVC, the relative contribution of each quadriceps muscle varies, with vastus intermedius contributing the most and vastus lateralis the least to the knee extension moment. Such findings indicate the subject- and condition-specific nature of load sharing among individual muscles. The influence of the dimension of each quadriceps muscle on the resulting knee extension, on the other hand, has not been demonstrated. The correlation between knee extensor moment and vastus lateralis PCSA was significant for isometric as well as isokinetic conditions (Blazevich et al., 2009). However, the relationship between the knee extension moment and PCSAs of other quadriceps muscles has not been studied yet.

Muscle size and its relationship with joint function become more intricate in the context of CP. Additional to the structural variations that occur due to size abnormalities in CP, it is also of interest to what extent they cause changes in a muscle’s capacity to generate force and functional deficits such as muscle weakness. Importantly, the impact of muscle size deficiency on muscle weakness is not uniform and it rather shows notable heterogeneity between joint movements and patients (Hanssen et al., 2021). The restricted ankle and knee joint ROMs in the patients compared to the healthy controls are evident (as exemplified in Table 2). However, intriguingly, no significant differences have been found in the peak ankle torque or knee moment between these groups (Barber et al., 2011a; Haberfehlner et al., 2016). This suggests that the overall torque-producing capacities of patients remain relatively constant, shedding light on the complex biomechanical basis of muscle weakness in CP.

Importantly, compared to their TD peers, children with CP are underpowered relative to their muscle size (Reid et al., 2015). The combined studies of intraoperative and MSK modeling indicated no strong relationship between MTU length and directly measured force amount in patients (Kaya et al., 2020; Kaya and Yucesoy, 2020). This is not so surprising since contributing factors to muscle strength (i.e., weakness) in children with CP are quite diverse. For example, the weakness in ankle plantar flexors has been explained with a smaller CSA but also the inability to fully activate them, as well as the co-activation of antagonists (Elder et al., 2003). Later findings in the quadriceps femoris and triceps surae supported that muscle weakness is accompanied by decreased agonist voluntary muscle activation as well as greater antagonist co-activation (Stackhouse et al., 2005). Such more pronounced antagonist co-activation in patients with CP can cause hampered movement due to increased joint stiffness (Unnithan et al., 1996; Damiano et al., 2000). Recent *in vivo* force measurements revealed how co-activation of the antagonistic muscles adversely affects the mechanical characteristics of a muscle to agree with the pathological knee joint condition in individuals with CP, e.g., for a primary knee flexor muscle, semitendinosus, in patients with CP, about 33% force increase was quantified due to the force transmission through the intermuscular interactions between activated synergists and antagonistic muscles (Ates et al., 2014; Ates et al., 2018b; Kaya et al., 2019; Kaya et al., 2020).

Despite muscle contractures being represented as the underlying reason for clinically observed restricted ROM, how they correlate with muscle weakness and eventual loss of function is very complex and not fully understood. However, consistent findings point to reduced muscle volume and PCSA, as well as compromised strength in individuals with CP (Elder et al., 2003; Poon and Hui-Chan, 2009; Barrett and Lichtwark, 2010). Understanding muscle weakness in CP also requires quantifying moment arm effects on in. In CP, the plantar flexor muscle weakness results from a combination of smaller plantar flexor muscle CSA and a shorter Achilles tendon moment arm compared to TD individuals (Kalkman et al., 2017). Moreover, a shortened moment arm of the muscle further compromises plantar flexion function (Gallinger et al., 2020): In CP, the triceps surae muscle moment arm is shorter than those in TD (by –6.5 mm). A shorter moment arm diminishes the mechanical advantage for the moment and contributes to a significantly lower peak moment (by 63%) measured in the patients during isometric MVCs. Coupled with a 52% less calculated muscle weakness compared to TD individuals, the shorter triceps surae moment arm apparently contributes to the reduced plantar flexion function in CP (Gallinger et al., 2020). Even in healthy populations, for the knee extensors, the longer the moment arm, the higher the torque-producing capacity (Hori et al., 2020); or for plantar flexors, the moment arm length is a predictor of decline in functional mobility (i.e., reduced velocity for walking at preferred speed) (Lee and Piazza, 2012). Considering the critical role of moment arms in force estimations in MSK modeling (Kalkman et al., 2017), these differences between groups must be analyzed and accounted for when studying CP-related muscle weakness and functional limitations. Besides, given the importance of MTU structures for generating muscle force, varying contributions of the changes in the fascicle, muscle, and tendon lengths to ROM may be important in determining the best treatment. Gaining insight into the extent to which different MTU structures contribute to joint rotation in individuals with CP is of paramount importance. In this regard, the medial gastrocnemius fascicles, for instance, lengthen 50% less in patients, regardless of ankle ROM (Barber et al., 2011a; Kalkman et al., 2018). When expressed as a percentage of MTU length change with joint position, relative muscle length change was smaller whereas relative tendon length change was larger in children with CP (Kalkman et al., 2018). Both muscle and tendon length changes are associated with ROM in children with CP and not in TD children, indicating that although the role of medial gastrocnemius in determining ankle ROM is not significant in healthy participants, it becomes significant in pathological conditions (Kalkman et al., 2018). Such adaptation should be considered when assessing and treating joint function in patients with CP, for example, during stretching exercises, the muscle may not be receiving targeted stretching stimulus with the applied length change (Kalkman et al., 2018). As another example, for hamstring muscles, understanding the subject-specific muscle length-joint function characteristics would have quite important clinical implications as one of the commonly used surgical techniques in CP is medial hamstring lengthening and this may cause serious side effects like a hyperextension of the knee or an increased anterior pelvic tilt (Kay et al., 2002; Adolfsen et al., 2007; Dreher et al., 2012). Smaller volume and PCSA of semitendinosus in children with CP evidently indicate muscle weakness (Haberfehlner et al., 2016). Muscle lengthening surgery aims at increasing the target muscle’s operational joint ROM (Zwick et al., 2002). However, since semitendinosus lengthening is presumed to induce even more weakness (Rutz et al., 2010), smaller muscles and shorter fascicles before surgery may be a risk factor for surgical side effects and may result in not a wider but narrower ROM (Haberfehlner et al., 2016). Basically, if the muscle belly is not sufficiently strained due to the lengthening of its tendon, fascicles that are already short before the surgery may get even shorter (Haberfehlner et al., 2016). Therefore, lengthening surgery potentially leads to further weakening in individuals with CP who already have weak muscles (Sätilä, 2020; Kaya Keles and Ates, 2022). Besides, keeping in mind that the paired force data for healthy individuals are missing, previous intraoperative testing indicated a wide enough operational joint ROM of semitendinosus in patients with CP (Ates et al., 2016; Ates et al., 2018b; Kaya et al., 2018; Kaya et al., 2020), which may question the necessity of muscle lengthening surgery as well. Still, this intervention may result in 1) shifts of the knee joint moment-angle or muscle force-angle curves towards more normal knee extension positions, 2) a decrease in the slope of these curves (i.e., lower stiffness of MTU structures), due to a longer tendon, and 3) lower fascial force transmission due to major discontinuity created in the intramuscular connective tissues during surgery (Yucesoy and Huijing, 2009; Ates et al., 2013). Therefore, hamstring lengthening requires highly critical decision-making, and botulinum toxin injections, among its main aims like diminishing spasticity and thereby improving joint function, can be a very useful tool for pre-operative testing of weakening of the muscles considered for lengthening surgery and so for filtering out patients who are at risk of deterioration after lengthening (Rutz et al., 2010).

### 4.2 Future directions

Today, imaging modalities have become the preferred tools for measuring muscle and tendon architecture, offering a range of advantages and disadvantages. Obtaining anthropometric features as well as MTU dimensions using those and combining the findings with joint torque assessments performed with a dynamometer allows us to search how the mechanics of individual MTUs influence joint functions. While these methods offer valuable information on muscle contracture and weakness observed in CP, a notable gap exists when it comes to relating the anthropometric features of MTUs to the power generated by a joint during functional activities (for example, increased passive stiffness of MTUs possibly due to shorter fascicles would lead to limitations in joint ROM in the patients). Many critical questions await exploration in future studies. Can specific MTU characteristics predict and quantify variations in joint function across different functional tasks? Which MTU contracture, observed in CP, most significantly contributes to muscle weakness and joint ROM limitations? How do dynamic changes in MTU length and force during functional movements influence joint kinetics and kinematics in individuals with CP? Furthermore, what longitudinal changes occur in MTU dimensions due to administered treatments or disease progression, and how do these changes relate to the alterations in the muscle’s force- and torque-generating capacity on the joint(s) it passes?

**FIGURE 2:**
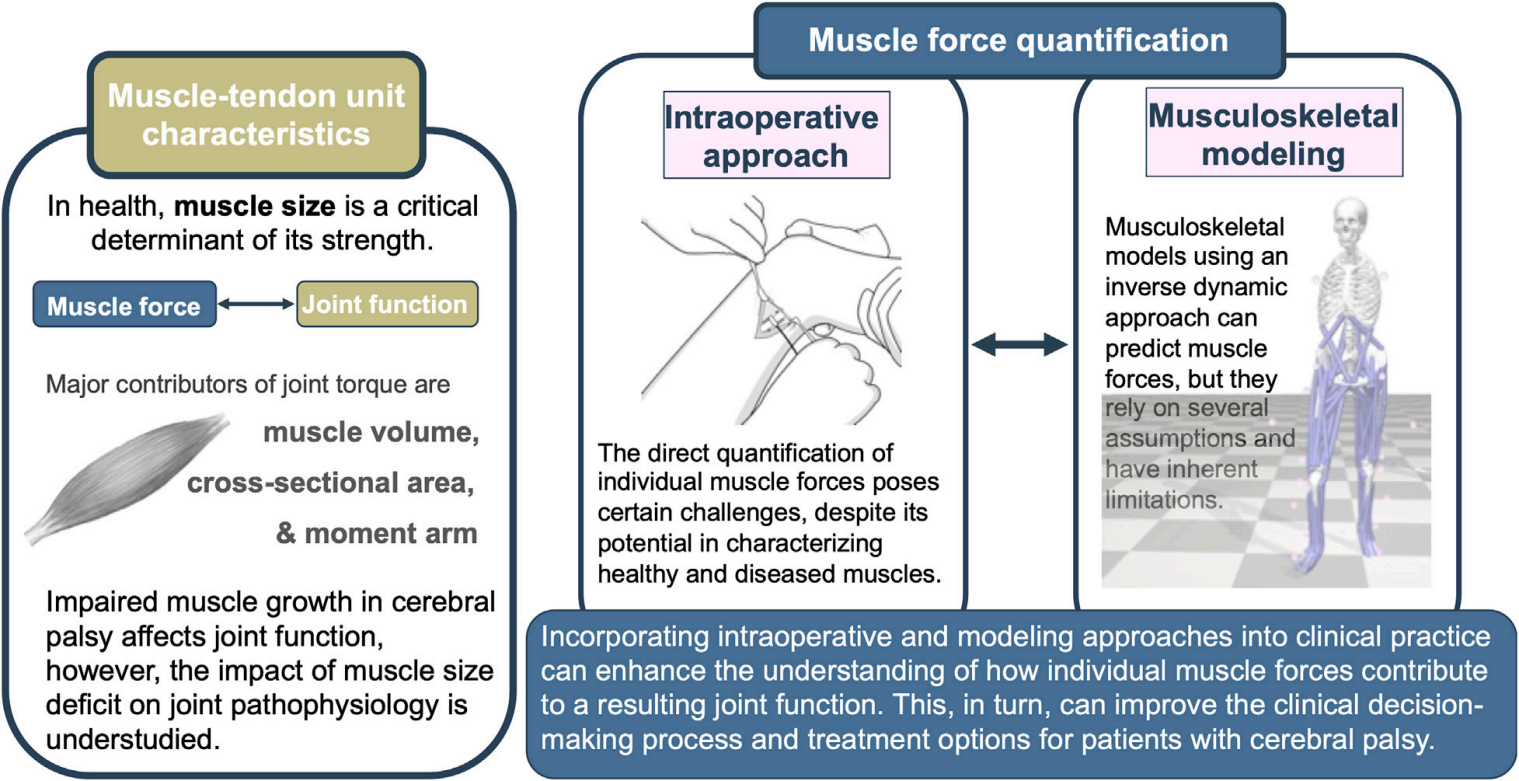
Graphical summary of the review depicting muscle-tendon unit characteristics, selected muscle force quantification techniques, and the potential benefits of their integration.

Over the past decade, significant progress has been made in methods for obtaining mechanical information at the muscular level. Two approaches stand out prominently (Figure 2). The intraoperative approach has emerged as a viable means of directly quantifying the force-generating capacity of muscles *in vivo*. This approach also enables the comparison of the passive and active state mechanics within a muscle, as well as relative to other muscles. Eliciting the overall muscle mechanical behavior, the intraoperative approach creates great potential for providing valuable new insights for understanding muscle mechanics. Revealing the underlying mechanisms of joint pathology observed in patients with CP could point to some special needs that should be focused on in the treatment.

Despite its merits, the intraoperative approach has notable limitations. Firstly, its invasive nature restricts its application to the selected surgical operations. Nonetheless, its successful application during correction surgeries for foot deformities, anterior cruciate ligament procedures, remedial surgery involving the release of a target muscle, or free-functioning muscle transfer surgery (e.g., Yucesoy et al., 2010; Ates et al., 2018b; Kaya et al., 2020; Persad et al., 2022) indicates its adaptability across diverse patient groups, with or without neuromuscular pathologies, facilitating data collection from various extremities.

Secondly, it captures forces in isometric conditions, as data collection during voluntary contractions is not feasible due to anesthesia. Consequently, the quantified forces may not fully represent dynamic loads or correspond directly to those experienced by the joint during daily activities. Instead, the maximum force achievable at the respective joint position is measured. This information, however, proves advantageous for comprehending various daily activities (e.g., walking, stair climbing, sit-to-stand, running, jumping, and more). To gain this insight, a 3D movement analysis of the patient is imperative, ideally coupled with simultaneous EMG, followed by the development of appropriate models. Once employed, the intraoperative approach offers a straightforward means of directly quantifying the functional capacity of the target MTU being tested. Despite the initial challenges posed by implementing the intraoperative technique into surgical routines, direct information on force characteristics holds significant scientific value 1) in terms of providing immediate and objective feedback for surgeons during operations and 2) clinically in the long run, through the development of many other techniques (e.g., MSK modeling) for better estimation of the muscle forces acting on each joint during various daily functions. Apart from the utilization of the intraoperative approach, modeling studies offer relatively more practical solutions.

Modeling studies have gained prominence over the years providing a means to predict the outcomes of some clinical treatments. For example, MSK modeling can be used to predict the acute outcome of interventions applied to patients with CP that may weaken their muscles even more (e.g., lengthening surgery, botulinum toxin injection, etc.). By estimating MTU length changes and muscle forces during certain activities, both the clinical decision-making and quantifying the treatment efficacy can be assisted. MSK modeling can be improved and designed to test how much the weakening of a target muscle alters the mechanics of other muscles and related joints (van der Krogt et al., 2012), to confirm the changes in MTU lengths and lengthening velocities (Salami et al., 2019), to compare the effects of treatments on muscle level activities and their force balance before and after the intervention (Wesseling et al., 2020), and thus, to predict which patients do benefit from the treatment (Arnold et al., 2006). However, MSK models are not without intrinsic limitations (e.g., Lin et al., 2012), particularly in the estimation of muscle forces which typically involving optimization, irrespective of the chosen strategy (inverse or forward dynamics-based).

The commonly employed inverse dynamics-based static optimization method relies on experimental capture of bone motion as model input, distributing joint torques among relevant muscles crossing the joint to reproduce observed motion (Erdemir et al., 2007; Röhrle et al., 2017). Yet, this approach lacks information on individual muscles’ load sharing, requiring minimization of a cost or objective function a fundamental intrinsic limitation (Erdemir et al., 2007). Forward dynamics-based dynamic optimization, on the other hand, uses an initial set of muscle activations as inputs to predict resulting motion based on muscle activity. Therefore, it necessitates muscle-level information as input (Röhrle et al., 2017). The iterative process involves comparing the solution against experimental data and updating muscle activations to best replicate experimental kinematics and, in some instances, kinetics. However, validating estimated muscle activation patterns often relies solely on comparing to EMG data (Erdemir et al., 2007), as experimentally obtained individual muscle forces are unavailable (Zajac et al., 2003). To improve this process, we propose the future use of intraoperatively measured muscle forces. Developing a modeling approach capable of offering insights into muscular load sharing, dynamic coupling, agonist-antagonist activity, energy transfer between joints through biarticular muscles, and force transfer between compartmental muscles through their connective tissue interactions (e.g., Zajac et al., 2002; Yucesoy, 2010) is essential. The direct measurement of muscle forces during surgeries and the augmentation of such a force database are crucial steps toward achieving this goal.

Importantly, the lack of patient-specific MRI and/or US data creates inaccuracies in the MSK model. Recognizing this, recent research has focused on enhancing the personalization of neuromusculoskeletal models by accurately reflecting morphological differences of MTU parameters (e.g., tendon slack length, optimal fiber length, the locations of muscle attachments, etc.) (Pellikaan et al., 2014; Pitto et al., 2019; Wesseling et al., 2019; Davico et al., 2022). Yet, considering the high inter-individual variances noted in the present review, it may not suffice to incorporate only a limited number of such features as input into the models. Another limitation regarding model-predicted muscle forces is that the moment arm and joint position predictions in MSK modeling are mainly based on muscle lengths. And MSK models generally take the muscles as independent entities that have connections to the bones only on their origin and insertion (distal and proximal ends). However, such estimations require focusing both on muscle size parameters and fascial interactions between muscles, other than the interaction at muscle ends alone. These indicate quite complex characteristics of muscles on force generation and the existence of mechanisms that should be included in the modeling which are not yet fully understood, even experimentally. MSK models serve as simplified representations of the musculoskeletal system, offering insights into overall biomechanics but potentially falling short of capturing detailed tissue-level mechanics. The concept suggesting that accurate estimates of muscle forces require computational models comprehensively representing both the skeleton and muscles (Erdemir et al., 2007) brings finite-element (FE) MSK models (Li et al., 2019; Navacchia et al., 2019; Li, 2021) to the forefront. FE models complement MSK modeling by providing a more granular examination of tissue mechanics. Initially, a rigid MSK model calculates muscle and joint loads at the whole-body level, and these loads serve as boundary conditions in a second model at the joint level to assess tissue deformation (Navacchia et al., 2019). FE models have the potential to offer a more comprehensive understanding of the interdependence between muscle forces and tissue deformation, improving subject-specific modeling. However, the fundamental question revolves around identifying the mechanical and physiological properties of MTUs that underlie the functioning of the MSK system and impact our ability to move. This requires a shift towards developing forward dynamics-based approaches over inverse dynamics. Describing a forward dynamics simulation framework of the musculoskeletal system based on continuum mechanical principles, a recent study successfully predicted realistic moment arms and muscle forces for the entire ROM of the upper limb (Röhrle et al., 2017). Yet, such studies remain limited. Regardless of the modeling method employed, direct validation of estimated muscle forces can only be achieved through *in vivo* data (Trinler et al., 2018). This is precisely the contribution expected from the intraoperative approach.

For these very reasons, it is necessary to carefully examine the muscle force data obtained directly from the experimental studies using the intraoperative approach and to continue to carry out similar studies in different groups of people and different muscles. A recent study (Brendecke et al., 2023), in which the triceps surae muscle group forces were measured using the intraoperative technique from the Achilles tendon in patients with non-neurological and neurological conditions, has been the first application of the intraoperative approach to the ankle joint. Interestingly, they reported lower passive forces in patients with idiopathic foot deformities than in patients with neurological conditions. In that study, the high inter-individual variability was also reported as its predecessors did at the knee joint suggesting the need for more comprehensive data collection before accurate modeling can be achieved.

In conclusion, while we have made strides in understanding the contribution of individual muscles to joint function, there is still considerable ground to cover. It is crucial to acknowledge potential sources of bias that may have influenced our review, necessitating careful consideration in future studies. The existing literature on the subject addressed in this article is not quite extensive, even among healthy individuals. Most studies predominantly explore either MTU characteristics or joint function in isolation, with quantitative investigations into the relationship between these factors notably scarce, particularly when involving patients with CP. The lack of standardized data collection methods, evident in the diverse torque measurement conditions and varied definitions of joint function parameters, presents a substantial obstacle to comprehensive assessments. To overcome these challenges, future studies should prioritize the direct measurement of muscle forces and the development of MSK models.

A promising future direction involves not only increasing the collection of experimentally acquired muscle force data, encompassing both patients and healthy individuals across different muscles but also the development and validation of mathematical models, such as MSK models and 3D muscle models. These models can be enhanced by incorporating personalized anthropometric features and MTU structural dimensions. Additionally, a holistic approach that considers the interactions between muscle size parameters and fascial connections between muscles, beyond mere muscle lengths, should be pursued. This multifaceted approach is essential for comprehending the complex mechanics of muscles in generating force related to joint functions.

Addressing the aforementioned questions can provide a more granular understanding of the intricate connections between MTU characteristics and joint function in CP, establishing implementation in clinical practice. Identifying the specific muscles responsible for abnormal joint function can significantly impact treatment planning. Evaluating the efficacy of interventions, such as physical therapy, surgery, or assistive devices, in enhancing MTU properties among individuals with CP is paramount. Emphasizing interventions targeting specific MTU characteristics can lead to more favorable outcomes in terms of joint power and ROM. Moreover, extending research to various populations with diverse musculoskeletal problems or focusing on specific MTU parameters holds promise for broader applications, including enhancing athlete performance through tailored exercise routines.
