# Supplementary material for: How mechanics of individual muscle-tendon units define knee and ankle joint function in health and cerebral palsy—a narrative review
Source: Front Bioeng Biotechnol. 2023 Dec 5;11:1287385. doi: 10.3389/fbioe.2023.1287385 (PMC10728775; doi:10.3389/fbioe.2023.1287385)
Supplement: Supplementary file 1 [file DataSheet1.PDF]

## Gracilis and semitendinosus muscles' forces with respect to the modeled muscle-tendon unit lengths in patients with cerebral palsy

Muscle-tendon unit length (MTU) is a key metric to monitor a muscle's ability to lengthen, hence estimating its changes is of interest, particularly in patients with cerebral palsy (CP). Because muscle length characteristics can affect its force-generation capacity, and hence its function, the identification of MTU length changes of commonly surgically treated muscles is relevant for obtaining a better operational outcome (e.g., after muscle lengthening surgery) in patients with CP.

The literature based on the use of the intraoperative approach, in which target muscles' forces are measured directly at their distal tendons and as a function of related joint angle, has revealed that spastic semitendinosus (Ates et al., 2016) and gracilis (Ates et al., 2013) produce their peak force in extended knee positions and only a limited portion of that is measured in flexed positions, in patients. However, how the force production of spastic muscles changes as a function of their MTU lengths has not been adequately demonstrated. Therefore, by combining the intraoperative technique with patients' musculoskeletal models, the relationship between the experimentally measured muscle forces and the modeled MTU lengths can be demonstrated in a patient-specific way. Previously, in patients with CP (age range: 6 to 13; mean age: 9 years 2 months (2 years 10 months); all are GMFCS level II), after conducting 3D gait analyses pre-surgically, then during surgery, gracilis and semitendinosus muscle forces were directly measured from their tendons both in the resting and the target muscle's selective activation conditions (i.e., representing the passive and active forces, respectively). The obtained muscle force-joint angle data were published in the literature as averaged muscle force graphs of the patient group (see Fig. 4 published in (Kaya et al., 2019) and Fig. 3 in (Kaya et al., 2020)). The MTU lengths corresponding to the joint positions from which muscle forces had been measured experimentally were then determined by musculoskeletal modeling (OpenSim) based on the individuals' 3D gait analysis data.

In the Table below, the given force values are based on the raw data that were previously published (Kaya et al., 2019, 2020). For each muscle in each patient, the first and last MTU lengths represent the shortest and longest MTU lengths corresponding to the combinations of the knee and hip joint positions tested intraoperatively. The muscle force data presented here can be used by normalizing them individually to the given anthropometric features of the patients.

**Table.** Muscle forces corresponding to MTU lengths in patients with CP.

| | Gracilis | | | Semitendinosus | | |
|---|---|---|---|---|---|---|
| **Patient/Anthropometric features** | **MTU length (cm)** | **Passive force (N)** | **Active force (N)** | **MTU length (cm)** | **Passive force (N)** | **Active force (N)** |
| (1) / Thigh length: 36.00 cm; mid-thigh circumference: 40.50 cm. | 38.70 | 0.00 | 62.02 | 40.80 | 0.54 | 36.16 |
| | 39.70 | 0.00 | 66.21 | 41.80 | 1.81 | 56.68 |
| | 40.70 | 0.00 | 72.76 | 42.80 | 1.04 | 64.63 |
| | 41.70 | 3.09 | 76.48 | 43.80 | 1.31 | 66.84 |
| | 42.70 | 8.51 | 75.89 | 44.80 | 4.50 | 68.12 |
| | 43.70 | 15.78 | 73.11 | 45.80 | 11.24 | 71.25 |
| | 44.65 | 24.07 | 73.69 | 46.80 | 20.98 | 77.00 |
| | | | | 47.80 | 31.91 | 84.11 |
| | | | | 48.80 | 41.04 | 89.27 |
| | | | | 49.80 | 44.12 | 87.18 |
| (2) | 38.66 | 32.84 | 91.99 | 40.80 | 1.56 | 18.98 |
| | 39.66 | 32.15 | 83.90 | 41.80 | 1.45 | 46.64 |
| | 40.66 | 31.22 | 91.63 | 42.80 | 2.69 | 58.42 |

| | | | | | | |
|---|---|---|---|---|---|---|
| / Thigh length: 36.50 cm; mid-thigh circumference: 39.00 cm. | 41.66 | 34.43 | 110.21 | 43.80 | 4.08 | 63.04 |
| | 42.66 | 42.36 | 134.67 | 44.80 | 5.14 | 66.30 |
| | 43.66 | 51.75 | 160.03 | 45.80 | 6.07 | 71.09 |
| | 44.61 | 55.64 | 180.42 | 46.80 | 7.78 | 77.37 |
| | | | | 47.80 | 11.85 | 82.19 |
| | | | | 48.80 | 20.55 | 79.68 |
| | | | | 49.80 | 36.87 | 61.06 |
| (3) / Thigh length: 30.00 cm; mid-thigh circumference: 32.50 cm. | 29.90 | 0.00 | 8.84 | 32.10 | 0.40 | 8.13 |
| | 30.90 | 1.91 | 27.38 | 33.10 | 0.56 | 9.34 |
| | 31.90 | 1.83 | 41.24 | 34.10 | 0.31 | 12.27 |
| | 32.90 | 3.00 | 48.31 | 35.10 | 0.65 | 16.12 |
| | 33.90 | 7.59 | 48.84 | 36.10 | 2.20 | 20.10 |
| | 34.90 | 16.31 | 45.43 | 37.10 | 5.29 | 23.41 |
| | 35.90 | 28.47 | 43.05 | 38.10 | 9.88 | 25.25 |
| | 36.00 | 29.80 | 43.13 | 39.10 | 15.62 | 24.84 |
| | | | | 40.10 | 21.82 | 21.36 |
| | | | | 40.50 | 24.20 | 18.93 |
| (4) / Thigh length: 31.00 cm; mid-thigh circumference: 36.00 cm. | 23.80 | 1.07 | 20.60 | 25.80 | 0.06 | 2.28 |
| | 24.80 | 1.03 | 21.36 | 26.80 | 0.93 | 4.44 |
| | 25.80 | 0.90 | 34.09 | 27.80 | 1.07 | 5.42 |
| | 26.80 | 1.04 | 46.69 | 28.80 | 1.07 | 6.77 |
| | 27.80 | 1.69 | 52.03 | 29.80 | 1.31 | 9.31 |
| | 28.80 | 2.92 | 48.03 | 30.80 | 1.97 | 13.06 |
| | 29.80 | 4.67 | 37.56 | 31.80 | 3.02 | 17.35 |
| | 30.22 | 5.52 | 32.97 | 32.80 | 4.26 | 20.71 |
| | | | | 33.80 | 5.24 | 20.96 |
| | | | | 34.60 | 5.45 | 16.96 |
| (5) / Thigh length: 31.00 cm; mid-thigh circumference: 35.80 cm. | 23.80 | 0.00 | 8.37 | 25.80 | 0.30 | 6.55 |
| | 24.80 | 0.45 | 16.08 | 26.80 | 0.17 | 2.75 |
| | 25.80 | 0.73 | 31.33 | 27.80 | 0.14 | 6.06 |
| | 26.80 | 2.19 | 44.38 | 28.80 | 0.11 | 10.44 |
| | 27.80 | 4.28 | 49.54 | 29.80 | 0.21 | 12.72 |
| | 28.80 | 6.44 | 45.23 | 30.80 | 0.77 | 12.62 |
| | 29.80 | 8.10 | 33.96 | 31.80 | 2.32 | 12.72 |
| | 30.22 | 8.52 | 28.58 | 32.80 | 5.60 | 18.50 |
| | | | | 33.80 | 11.56 | 38.29 |
| | | | | 34.60 | 19.01 | 71.67 |
| (6) / Thigh length: 27.00 cm; mid-thigh circumference: 29.20 cm. | 26.70 | 0.04 | 25.42 | 28.80 | 2.63 | 9.61 |
| | 27.70 | 0.17 | 42.29 | 29.80 | 2.46 | 14.26 |
| | 28.70 | 0.11 | 62.54 | 30.80 | 2.25 | 19.99 |
| | 29.70 | 0.85 | 80.83 | 31.80 | 2.32 | 24.10 |
| | 30.70 | 2.77 | 91.83 | 32.80 | 2.98 | 25.25 |
| | 31.70 | 5.67 | 90.22 | 33.80 | 4.55 | 23.41 |
| | 31.85 | 6.14 | 88.55 | 34.80 | 7.35 | 19.88 |
| | | | | 35.80 | 11.70 | 17.27 |
| | | | | 36.10 | 13.35 | 17.22 |
| (7) / Thigh length: 35.00 cm; mid-thigh circumference: 43.00 cm. | 31.50 | 12.37 | 23.70 | 34.00 | 7.21 | 22.92 |
| | 32.50 | 9.55 | 47.86 | 35.00 | 7.17 | 48.19 |
| | 33.50 | 10.43 | 49.89 | 36.00 | 7.18 | 65.41 |
| | 34.50 | 13.06 | 51.19 | 37.00 | 7.21 | 68.82 |
| | 35.50 | 15.48 | 59.60 | 38.00 | 7.23 | 67.20 |
| | 36.50 | 15.73 | 69.40 | 39.00 | 7.26 | 65.89 |
| | 37.50 | 11.85 | 61.34 | 40.00 | 7.30 | 66.79 |
| | 38.06 | 7.15 | 37.36 | 41.00 | 7.36 | 68.41 |
| | | | | 42.00 | 7.49 | 65.79 |
| | | | | 43.00 | 7.72 | 50.56 |
| | | | | 43.10 | 7.75 | 47.92 |
| | | | | 43.20 | 7.79 | 45.02 |

| | | | | | | |
|---|---|---|---|---|---|---|
| (8) / Thigh length: 37.00 cm; mid-thigh circumference: 37.00 cm. | 34.90 | 8.51 | 34.09 | 37.40 | 7.50 | 9.46 |
| | 35.90 | 8.80 | 48.45 | 38.40 | 7.45 | 10.58 |
| | 36.90 | 8.45 | 54.17 | 39.40 | 7.46 | 11.17 |
| | 37.90 | 8.51 | 58.21 | 40.40 | 7.48 | 12.15 |
| | 38.90 | 9.48 | 63.67 | 41.40 | 7.49 | 13.96 |
| | 39.90 | 11.25 | 69.81 | 42.40 | 7.47 | 16.50 |
| | 40.90 | 13.14 | 71.99 | 43.40 | 7.45 | 19.19 |
| | 41.36 | 13.72 | 69.49 | 44.40 | 7.45 | 20.91 |
| | | | | 45.40 | 7.49 | 20.08 |
| | | | | 46.40 | 7.65 | 14.57 |
| (9) / Thigh length: 38.00 cm; mid-thigh circumference: 39.50 cm. | 34.90 | 5.47 | 12.18 | 37.40 | 8.74 | 8.89 |
| | 35.90 | 6.16 | 36.96 | 38.40 | 8.69 | 23.33 |
| | 36.90 | 5.78 | 52.86 | 39.40 | 8.72 | 31.38 |
| | 37.90 | 5.79 | 61.69 | 40.40 | 8.77 | 35.76 |
| | 38.90 | 6.90 | 65.22 | 41.40 | 8.80 | 38.24 |
| | 39.90 | 9.11 | 65.25 | 42.40 | 8.81 | 39.66 |
| | 40.90 | 11.66 | 63.57 | 43.40 | 8.80 | 39.95 |
| | 41.36 | 12.57 | 62.72 | 44.40 | 8.82 | 38.08 |
| | | | | 45.40 | 8.93 | 32.12 |
| | | | | 46.40 | 9.22 | 19.18 |
| (10) / Thigh length: 26.00 cm; mid-thigh circumference: 28.00 cm. | 23.50 | 36.82 | 18.63 | 25.80 | 4.90 | 22.94 |
| | 24.50 | 36.80 | 23.95 | 26.80 | 4.24 | 22.93 |
| | 25.50 | 36.79 | 35.95 | 27.80 | 4.19 | 24.03 |
| | 26.50 | 33.93 | 44.85 | 28.80 | 4.91 | 23.76 |
| | 27.50 | 26.42 | 48.28 | 29.80 | 6.55 | 21.29 |
| | 28.50 | 13.55 | 51.30 | 30.80 | 9.28 | 17.47 |
| | 28.99 | 5.36 | 56.10 | 31.80 | 13.26 | 14.87 |
| | | | | 32.80 | 18.64 | 17.68 |

## References


Ates, F., Temelli, Y., and Yucesoy, C. A. (2013). Human spastic Gracilis muscle isometric forces measured intraoperatively as a function of knee angle show no abnormal muscular mechanics. *Clinical Biomechanics* 28, 48–54. doi: 10.1016/j.clinbiomech.2012.08.012.

Ates, F., Temelli, Y., and Yucesoy, C. A. (2016). The mechanics of activated semitendinosus are not representative of the pathological knee joint condition of children with cerebral palsy. *Journal of Electromyography and Kinesiology* 28, 130–6. doi: 10.1016/j.jelekin.2016.04.002.

Kaya, C. S., Bilgili, F., Akalan, N. E., Temelli, Y., Ateş, F., and Yucesoy, C. A. (2019). Intraoperative experiments combined with gait analyses indicate that active state rather than passive dominates the spastic gracilis muscle's joint movement limiting effect in cerebral palsy. *Clinical Biomechanics* 68, 151–157. doi: 10.1016/j.clinbiomech.2019.06.005.

Kaya, C. S., Bilgili, F., Akalan, N. E., and Yucesoy, C. A. (2020). Intraoperative testing of passive and active state mechanics of spastic semitendinosus in conditions involving intermuscular mechanical interactions and gait relevant joint positions. *J Biomech* 103, 10955. doi: 10.1016/j.jbiomech.2020.109755.
